# Optimizing bowel preparation for colonoscopy: A cross-sectional study of the Chinese population

**DOI:** 10.3389/fpubh.2022.953441

**Published:** 2022-08-12

**Authors:** Li Luo, Yuan Liu, Lingling Zhang, Yihuan Lai, Yansheng Li, Kejia Liu, Houwu Gong, Dapeng Jiang, Erchuan Wang

**Affiliations:** ^1^Department of Gastroenterology, West Hospital, Union Hospital, Tongji Medical College, Huazhong University of Science and Technology, Wuhan, China; ^2^Cancer Center, Union Hospital, Tongji Medical College, Huazhong University of Science and Technology, Wuhan, China; ^3^Department of Gastroenterology, West Hospital, Union Hospital Affiliated to Tongji Medica, Wuhan, China; ^4^DHC Mediway Technology Co., Ltd., Medical Big Data Research Institute, Beijing, China

**Keywords:** bowel preparation, colonoscopy, Ottawa Bowel preparation Scale, age, quality of bowel preparation

## Abstract

**Background:**

The quality of bowel preparation is an important factor in the success of colonoscopy. However, the quality of bowel preparation is often affected by multiple factors. The main objective of this study was to explore the specific factors that affect the quality of bowel preparation.

**Methods:**

Patients were consecutively recruited from the gastroenterology department in Union Hospital, Tongji Medical College, Huazhong University of Science and Technology in Wuhan from May 2018 to December 2018. All patients were undergoing colonoscopy. Bowel preparation was evaluated by the Ottawa Bowel preparation Scale (OBPS) and all patients were categorized into 2 groups according to the OBPS. Multivariate analysis was conducted to identify the factors associated with bowel preparation quality.

**Results:**

A total of 910 patients were included in the analysis with an average age of 48.62 ± 13.57 years. Patient source (*P* < 0.001) and the preparation method (*P* = 0.029) were correlated with OBPS adequacy. In addition, after stratified by age, preparation method (*P* = 0.022) was a significant factor among patients under 50 years old; whereas waiting time (*P* = 0.005) was a significant factor among patients over 50 years old.

**Conclusion:**

Bowel preparation should be tailored based on the age of the patients to determine the most appropriate plan, including the most appropriate waiting time and the most appropriate purgative combination. Doctors should also focus more on the quality of bowel preparation in inpatients, who are more likely than outpatients to have an inadequate bowel preparation.

## Introduction

Colonoscopy is the preferred procedure for investigating large-bowel and terminal ileal disease in patients with digestive system disorders (1, 2). Given the high diagnostic sensitivity and specificity, colonoscopy had become the gold standard for colorectal adenoma and carcinoma among the populations at high risk (3). To observe bowel mucosa clearly for endoscopists, optimal bowel preparation is essential to achieve a high-quality colonoscopy (4, 5). However, the quality of bowel preparation is often affected by many factors, such as waiting time (the time between the last laxative administration and the beginning of colonoscopy), type and method of administration of laxative, patient compliance, etc. An inadequate bowel preparation regimen is an important factor that affects the adequacy of bowel preparation. It is reported that the rate of inadequate bowel preparation ranges from 20 to 30% (6). Therefore, it is crucial to improve the preparation adequacy rate.

A variety of different bowel preparation methods such as simethicone, mannitol, polyethylene glycol electrolyte (PEG) solution, and colonoscopy bowel capsule are available to be used to clean the bowel (7–9). However, as multiple factors may influence the preparation quality, none of the above bowel preparation methods has achieved satisfactory quality for both the clinician and the patient. It had been reported that patients' factors such as age, sex, education level, personal preference, and income status may be associated with the quality of bowel preparation (1, 10). In addition, as severe electrolyte disturbances may occur, the latest international guideline also recommends that the preparation protocol, especially the effectiveness of split dose preparation and adding bisacodyl or Senna to the standard preparation are also pivotal to good bowel preparation (11, 12). Yadav et al. reported that split-dose polyethylene glycol (PEG) was superior to single-dose PEG for patient compliance and side effects (13).

Nowadays, a lot of studies had reported the importance of preparation protocol in bowel preparation; however, the conclusions lack conformity (14, 15). Apart from the existing evidence, this study aimed to assess the variability of bowel preparation regimes employed within the inpatient and outpatient, and to find the correlation between different methods and the adequacy of bowel preparation before colonoscopy.

## Methods

### Patients enrolled

This was a single-center cross-sectional study. Patients were consecutively recruited from the gastroenterology department in Union Hospital, Tongji Medical College, Huazhong University of Science and Technology in Wuhan from May 2018 to December 2018. All patients were undergoing colonoscopy. Patients with allergies to the bowel preparations used, suspected or diagnosed with bowel obstruction, infectious disease, previous bowel preparation in the past 14 days, decompensated heart failure, severe acute renal failure, severe liver disease, or severe electrolyte imbalance were excluded from this study. The study was approved by the Ethics Committee of Union Hospital and conducted in accordance with the Declaration of Helsinki. Written informed consent forms were obtained from all participants.

All patients enrolled accepted one of the bowel preparation methods as follows according to the Chinese guideline for bowel preparation for colonoscopy (16). Briefly, bowel preparation methods were as follows: 1. Mannitol (Preparation method 1); 2. Three packs of Fu Jing Qing (Polyethylene Glycol Electrolytes), one pack at eight o 'clock the night before, one pack at four o 'clock in the morning, and one pack at five o 'clock (Preparation method 2); 3. Three packs of Fu Jing Qing (same time as above) and 1 bottle of Simeticone (Preparation method 3). The quality of bowel preparation was evaluated by the Ottawa Bowel preparation Scale, and those with OBPS ≤ 7 points were considered as qualified intestinal preparation (OBPS, 0–14 points, the higher the score, the worse the quality of bowel preparation) (7). All patient demographics including age, sex, source, preparation method, wait time, and patient self-assessment were extracted manually from electronic medical records.

### Statistics

The baseline characteristics of participants with adequate or inadequate OBPS were compared using the chi-square test for categorical variables and the two-sample *t*-test for continuous variables. A *P*-value of < 0.05 was considered statistically significant. Odds ratios (OR) and 95% confidence intervals (CI) for the association between OBPS adequacy and potential risk factors were estimated using the multivariable logistic regression model. An OR>1 is associated with a higher Ottawa score, and consequently, poorer bowel preparation. The association was further analyzed in different age groups using the multivariable logistic regression model. All statistical analyses were performed using R software, version 4.1.1.

## Results

### Basic characteristic

**Figure 1:**
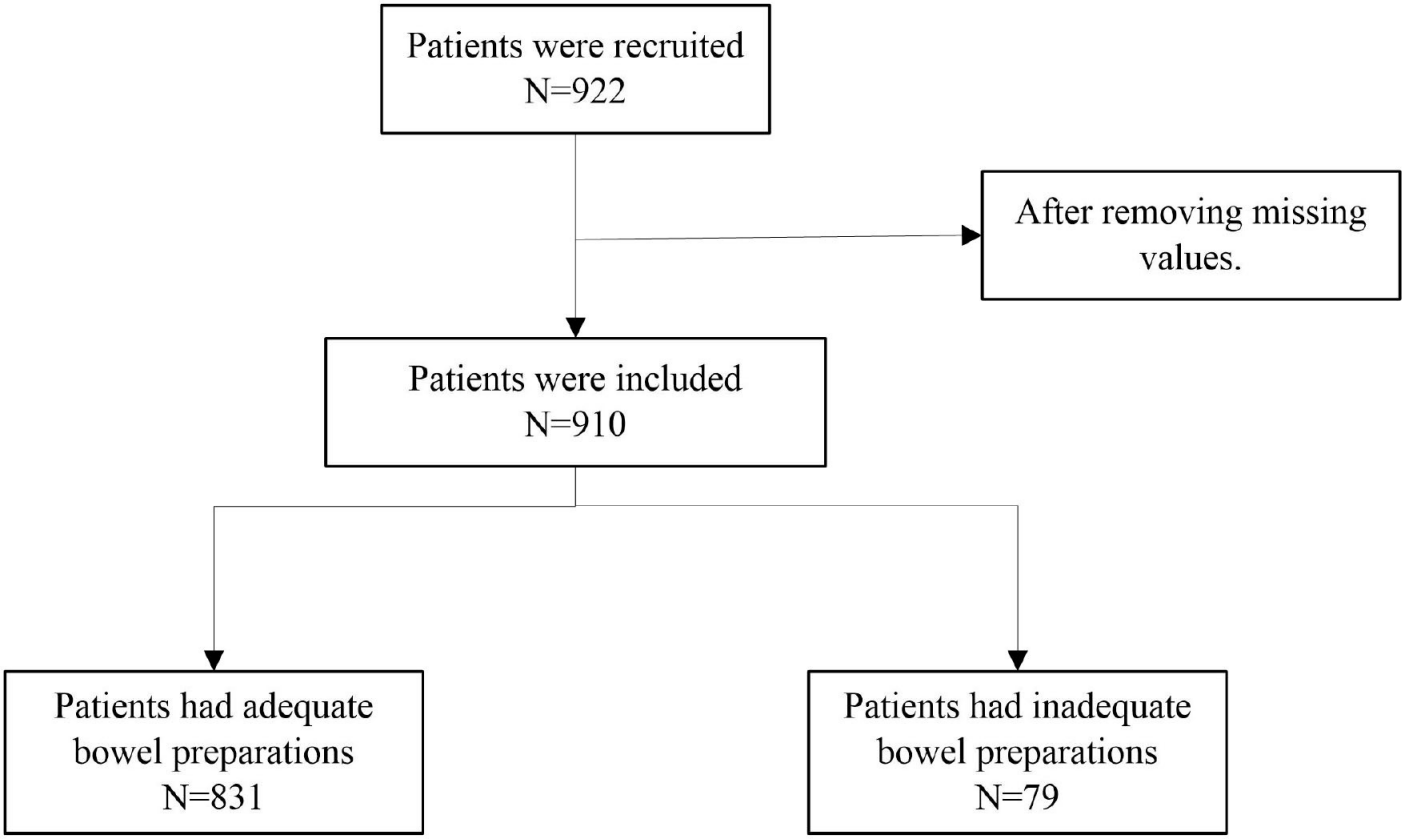
Flow chart of patients included in the experiment.

A total of 922 patients were recruited, and finally, 910 patients were included in the analysis after removing missing values, as shown in Figure 1. The average age of the patients was 48.62 ± 13.57 years. Among them, 831 had adequate bowel preparations, with an average age of 48.47 ± 13.54 years. A total of 79 patients had inadequate bowel preparations, with an average age of 50.24 ± 13.87 years. Of all patients, 363 were from the inpatient unit and 547 were from the outpatient unit. A total of 315 (37.91%) patients from the inpatient care and a total of 516 (62.09%) patients from the outpatient care had got adequate preparations. In addition, 672 (80.87%) patients who got adequate preparations and 61 (77.22%) patients who got inadequate preparations received the third preparation method. The baseline characteristics of patients stratified by preparation adequacy were summarized in Table 1.

**Table 1:** Baseline characteristics of the OBPS.

**Variable**	**All (*n* = 910)**	**Adequate (*n* = 831)**	**Inadequate (*n* = 79)**	***P* Value**
Age (yrs)	48.62 ± 13.57	48.47 ± 13.54	50.24 ± 13.87	0.279
Gender (male)	555 (60.99)	505 (60.77)	50 (63.29)	0.661
Source				
Inpatient	363 (39.89)	315 (37.91)	48 (60.76)	<0.001
Outpatient	547 (60.11)	516 (62.09)	31 (39.24)	
Preparation method				
One	69 (7.58)	63 (7.58)	6 (7.59)	0.630
Two	108 (11.87)	96 (11.55)	12 (15.19)	
Three	733 (80.55)	672 (80.87)	61 (77.22)	
Waiting time (h)				
<4	102 (11.21)	96 (11.55)	6 (7.59)	0.186
(4–6)	311 (34.18)	290 (34.90)	21 (26.58)	
(6–8)	360 (39.56)	324 (38.99)	36 (45.57)	
≥ 8	137 (15.05)	121 (14.56)	16 (20.25)	
Patient self-evaluation score	3.78 ± 0.44	3.78 ± 0.43	3.73 ± 0.50	0.400

### The relationship between the OBPS and the risk factors

Briefly, a total of 831 patients were qualified according to the OBPS. After conducting a univariate analysis of patients' age, gender, source (inpatient/outpatient), preparation method, waiting time, and self-assessment with the OBPS adequacy rate, we found that the effect of patient source on the OBPS adequacy rate was statistically significant (*P* < 0.001).

The results of the multivariate analysis of the OBPS adequacy rate were shown in Table 2. After further integrating all the above factors into the logistic regression model, we found that the source (P < 0.001) had statistically significant effects on whether the OBPS was adequate. The results showed that inpatients were more likely to have inadequate OBPS than outpatients (OR = 3.18). We also found that the preparation method (*p* = 0.029) had significant effects on whether the OBPS was adequate. The results showed that patients who used the first preparation method were more likely to have inadequate OBPS than patients who used the third preparation method (OR = 2.27).

**Table 2:** Multivariate analysis of factors associated with the OBPS.

**Variable**	**Odds ratio**	**95% CI**	***P* Value**
Age (yrs)	1.00	0.98–1.02	0.750
Gender (male)	1.24	0.76–2.05	0.397
Source (inpatient)	3.18	1.83–5.66	<0.001
Preparation method			
Three	1 (Reference)		
One	2.27	1.05–4.65	0.029
Two	0.77	0.28–1.75	0.559
Waiting time (h)			
(4–6)	1 (Reference)		
<4	0.91	0.32 – 2.23	0.844
(6–8)	1.55	0.88 – 2.78	0.133
≥8	1.44	0.70 – 2.88	0.313
Patient self-evaluation	0.73	0.44 – 1.25	0.232
score			

### Multivariate analysis: The relationship between OBPS adequacy and risk factors in patients less than or over 50 years old

The results of the multivariate analysis of the OBPS adequacy rate stratified by age were shown in Table 3. Age, sex, source, preparation plan, waiting time, and patient self-assessment results were incorporated into the logistic regression model (<50 years or ≥ 50 years). It was found that source (*P* = 0.001) and preparation method (*P* = 0.022) had significant effects on whether the OBPS was adequate for patients who were < 50 years old. The results showed that inpatients were more likely than outpatients to have an inadequate OBPS (OR = 3.79); and patients who used preparation method 1 were more likely to have an inadequate OBPS than patients who used preparation method 3 (OR = 3.05). For those patients who were older than or equal to 50 years old, patient source (*P* = 0.005), waiting time 6–8 h (*P* = 0.005), and patient self-assessment (*P* = 0.039) had significant effects on OBPS adequacy. The results showed that inpatients were more likely to have an inadequate OBPS than outpatients (OR = 3.15); patients who waited between 6 and 8 h were more likely to have an inadequate OBPS than patients who waited between 4 and 6 h (OR = 3.65); and patients who had a higher self-assessment score were more likely to have an adequate OBPS (OR = 0.41).

**Table 3:** Multivariate analysis of factors associated with the OBPS segmented by age.

**Subgroup**	**Variable**	**Odds ratio**	**95% CI**	***P* Value**
Age group (< 50 years)	Age (years)	1.00	0.95–1.04	0.914
	Gender (male)	0.86	0.42–1.84	0.686
	Source (inpatient)	3.79	1.71–8.70	0.001
	Preparation method			
	Three	1 (Reference)		
	One	3.05	1.13–7.88	0.022
	Two	0.32	0.02–1.68	0.281
	Waiting time (h)			
	(4–6)	1 (Reference)		
	<4	0.41	0.06–1.66	0.271
	(6–8)	0.66	0.28–1.52	0.330
	≥8	1.27	0.48–3.21	0.615
	Patient self-evaluation score	0.96	0.50–1.99	0.911
Age group (≥ 50 years)				
	Age (years)	1.01	0.97–1.06	0.555
	Gender (male)	1.72	0.88–3.45	0.117
	Source (inpatient)	3.15	1.45–7.31	0.005
	Preparation method			
	Three	1 (Reference)		
	One	1.79	0.47–5.66	0.346
	Two	1.12	0.35–3.01	0.829
	Waiting time (h)			
	(4–6)	1 (Reference)		
	<4	2.07	0.51–7.40	0.273
	(6–8)	3.65	1.57–9.62	0.005
	≥8	1.66	0.53–5.15	0.374
	Patient self-evaluation score	0.41	0.18–1.00	0.039

## Discussion

The effectiveness of a colonoscopy depends on adequate bowel preparation (17); however, adequate bowel preparation is a complex process that is influenced by several factors. In this study, we proposed a single-center cross-sectional study to evaluate the risk factors that may influence bowel preparation. The results showed that patient source (*P* < 0.001) and the preparation method (*P* = 0.029) were important risk factors that might influence the quality of bowel preparation. This was in accordance with what cao et al. had reported in a meta-analysis (9). Furthermore, after stratified by age, preparation method (*P* = 0.022) was a significant factor among patients under 50 years old; whereas waiting time (*P* = 0.005) was a significant factor among patients over 50 years old.

Due to its high diagnostic sensitivity and specificity, colonoscopy has become the gold standard for colorectal cancer screening (18). The quality of a colonoscopy depends on adequate visualization, which relies on the quality of bowel cleaning. It has been shown that up to 26% of adenomas are missed by standard colonoscopy. This missing rate could be decreased by adequate bowel preparation and auxiliary techniques (19). Thus, it is important to choose a suitable method according to the patient's self-physical condition before the colonoscopy (20). Seo et al. found that the time interval between the last dose of the agent and the start of colonoscopy is one of the important factors to determine satisfactory bowel preparation quality (21). Ray-Offor et al. have reported that the educational status of patients is a strong risk factor associated with inadequate bowel preparation for colonoscopy in the Nigerian population (22). Therefore, it is necessary to further explore the factors that may influence bowel preparation quality.

In our study, we found that inpatients were more likely than outpatients to have inadequate bowel preparation (OR = 3.18). The reason for the result might be that inpatients have a more serious disease than outpatients. We also found that patients who used the first preparation method were more likely to have inadequate OBPS than patients who used the third preparation method (OR = 2.27). In addition, after stratified by age, the preparation method was a significant predictor for the OBPS adequacy among patients under 50 years old; while waiting time was a significant predictor among patients over 50 years old. For patients under 50 years old, those who used preparation method 1 were more likely to have an inadequate bowel preparation than those who used the preparation method 3 (OR = 3.05); and for patients over 50 years old, those who waited between 6 and 8 h before colonoscopy were more likely to have an inadequate bowel preparation than those who waited between 4 and 6 h before colonoscopy (OR = 3.65). Our study was in accordance with what had been reported by other researchers (23–25).

This study has several limitations. First, it was conducted at a single center and was a single-arm, retrospective study, which may cause bias of the results. Second, it has demonstrated that patient-related factors like drugs intake, previous abdominal surgery, and chronic constipation may be associated with an increased risk of inadequate preparation; nevertheless, we did not enroll these factors, which may influence the accuracy of the results (26–29). Third, past research showed that poor patient compliance may also cause inadequate bowel preparation (30). Educating and motivating patients to improve compliance could obtain better bowel cleansing; however, our study did not evaluate such aspect. Last but not least, tolerability is a major factor in good bowel preparations. Most patients that use laxatives to promote bowel preparation may experience nausea, vomiting, and other adverse reactions, which may also influence the quality of bowel preparation.

## Conclusion

In conclusion, we believe that bowel preparation should be tailored based on the age of the patients to determine the most appropriate plan, including the most appropriate waiting time and the most appropriate purgative combination. In addition, the results of our study showed that doctors should focus more on the quality of bowel preparation in inpatients, who are more likely than outpatients to have inadequate bowel preparation.

## Data availability statement

The original contributions presented in the study are included in the article/supplementary material, further inquiries can be directed to the corresponding author.

## Ethics statement

Ethical review and approval was not required for the study on human participants in accordance with the local legislation and institutional requirements. Written informed consent for participation was not required for this study in accordance with the national legislation and the institutional requirements. Ethical review and approval was not required for the animal study because this project is a retrospective study.

## Author contributions

LL wrote the manuscript. LL, EW, YLiu, and LZ collected the data. YLi, KL, YLai, HG, and DJ assisted with data statistics and interpretation. All authors have read and approved the final version of the manuscript.

## Conflict of interest

Authors YLi, KL, YLai, HG, and DJ were employed by DHC Mediway Technology Co., Ltd. The remaining authors declare that the research was conducted in the absence of any commercial or financial relationships that could be construed as a potential conflict of interest.

## Publisher's note

All claims expressed in this article are solely those of the authors and do not necessarily represent those of their affiliated organizations, or those of the publisher, the editors and the reviewers. Any product that may be evaluated in this article, or claim that may be made by its manufacturer, is not guaranteed or endorsed by the publisher.
